# Mapping and Quantification of Non-Coding RNA Originating from the rDNA in Human Glioma Cells

**DOI:** 10.3390/cancers12082090

**Published:** 2020-07-28

**Authors:** Anastasia A. Sadova, Natalia S. Kupriyanova, Galina V. Pavlova

**Affiliations:** 1 Academic Chair of Biochemistry and Molecular Biology, Faculty of General Medicine, Pirogov Russian National Research Medical University, 117997 Moscow, Russia; 2 Lab. of Genome Organization, Institute of Gene Biology, Russian Academy of Sciences, 119334 Moscow, Russia; kupriyanova-45@mail.ru; 3 Lab. of Neurogenetics and Genetics Development, Institute of Gene Biology, Russian Academy of Sciences, 119334 Moscow, Russia; lkorochkin@mail.ru; 4 Institute of Molecular Medicine, Sechenov First Moscow State Medical University, 119991 Moscow, Russia; 5 Department of X-ray and radioisotope diagnostic methods, Burdenko Neurosurgical Institute, 125047 Moscow, Russia

**Keywords:** non-coding RNA, pRNA, human rDNA, glioma, IGS

## Abstract

Ribosomal DNA is one of the most conserved parts of the genome, especially in its rRNA coding regions, but some puzzling pieces of its noncoding repetitive sequences harbor secrets of cell growth and development machinery. Disruptions in the neat mechanisms of rDNA orchestrating the cell functioning result in malignant conversion. In cancer cells, the organization of rRNA coding genes and their transcription somehow differ from that of normal cells, but little is known about the particular mechanism for this switch. In this study, we demonstrate that the region ~2 kb upstream of the rDNA promoter is transcriptionally active in one type of the most malignant human brain tumors, and we compare its expression rate to that of healthy human tissues and cell cultures. Sense and antisense non-coding RNA transcripts were detected and mapped, but their secondary structure and functions remain to be elucidated. We propose that the transcripts may relate to a new class of so-called promoter-associated RNAs (pRNAs), or have some other regulatory functions. We also hope that the expression of these non-coding RNAs can be used as a marker in glioma diagnostics and prognosis.

## 1. Introduction

### 1.1. The Intergenic Spacer of rDNA Harbors Many Intriguing Features

Human rDNA codes for three types of rRNA molecules, which constitute the essential component of eukaryotic ribosomes. The genes are located on five human acrocentric chromosomes forming clusters in “head-to-tail” orientation, the coding sequences being separated by the ~30 kb intergenic spacer (IGS) [1]. Although for many years it was thought that this huge part of rDNA is nonsense and inert, recent studies have demonstrated that IGS is in fact a cell management and control center since it harbors regulatory sequences defining the vital processes in a particular cell [2]. This region is known to contain Alu-repeats and microsatellites, some of which are rather conserved among humans and other apes [3]; cdc27 pseudogene and multiple c-Myc binding sites characteristic to apes and other mammals also present throughout the human IGS [4].

For more than two decades, it has been known that the biggest part of ribosomal genes is transcribed in vertebrates [5,6]. The zones in the human IGS enriched with conserved regions are transcribed in many cell lines, and the conservative sequences themselves are associated with enhancers or transcription start sites [4]. It has been demonstrated, that two non-coding RNA molecules named IGS21RNA and IGS28RNA are transcribed under stress conditions from corresponding regions of ribosomal intergenic spacer in human cell lines to immobilize proteins and thus regulate their functions at the posttranslational level [7]. Further, a microRNA hsa-mir-6724 homologous to the sequence of the pre-promoter region of IGS was predicted during the investigation of small non-coding RNAs (sncRNAs) in the adult human epididymis [8]. In addition, a short promoter RNA (pRNA) originating from the ‘tails’ of murine rDNA repeats and transcribed with the help of a spacer promoter was shown not only to regulate the expression of rRNAs, but also to participate in nucleolus organization and cell differentiation [9,10]. A deconvolution protocol for ChIP-seq data revealed the sequence homologous to the Mus musculus spacer promoter and to the promoter of 45S rRNA genes in humans [11]. Human pRNA was predicted by computational methods [12], however, it is not likely to be synthesized in the corresponding area as the predicted human spacer promoter lies upstream of the sequence coding for the predicted microRNA hsa-mir-6724 [7]. Interestingly, the region upstream of the predicted spacer promoter is transcribed in human lung cancer cell lines [13], inspiring the search for another spacer promoter.

In the current work we map and estimate the expression of non-coding transcripts originating from the pre-promoter region of rDNA upstream of the predicted spacer promoter in several human cell lines including immortalized cultures of glioma and glioblastoma, and non-cancer cells.

### 1.2. Glioma and Glioblastoma Need Deep Molecular Phenotype Characterization

Glioma, or anaplastic astrocytoma, comprises nearly one third of central nervous system tumors diagnosed and most high-grade brain tumors belong to this specific type, with poor prognosis and high mortality rates [14]. In this regard, it is of great importance to find markers and approaches for early diagnosis of the malignant conversion and tools for proper life prognosis in patients with glioma. A recent meta-analysis of one class of small non-coding RNAs—microRNAs—indicated that they could be successfully used as diagnostic biomarkers for glioma, especially when taken as panels of multiple miRNAs, as individual miRNAs may show high heterogeneity, specificity and sensitivity [15]. MicroRNAs differences in the expression profiles of healthy and malignant cells may be based on altered processing of precursor miRNAs and the overall maturation process of a particular microRNA. Interestingly, the deregulation of genes involved in miRNA maturation strongly correlates with survival rates in patients diagnosed with glioma [16].

Tumors diagnosed as glioma fall into several groups according to their etiology, prognosis and response to therapy due to the variety in their individual molecular-genetic characteristics [17]. For these reasons, high-grade gliomas are usually insensitive to monotherapy, and tumors recur, therefore it is of great importance to develop next generation molecular therapeutic strategies employing gene therapy, oncogenic microRNA targeting, cancer immunotherapy, etc [18].

Among the World Health Organization (WHO) grades of astrocytoma, glioblastoma is classified as being the most progressed and severe of brain tumors (grade IV). Glioblastoma is one of the most aggressive human brain cancers with the prognosis of survival less than 15 months [19], and various existing treatment approaches can only modestly reduce the risk of recurrence after therapy [20].

Since some microRNAs are shown to have an expression profile in glioblastoma different from normal tissues, these molecules, most of which are associated with modulation of oncogenes and tumor suppressors, have great potential in developing anticancer targeted therapies [21]. MicroRNAs can be both up-regulated and down-regulated, and even have a variable regulation level in glioblastoma cells, suggesting that an integrative orchestra contributes to a tumor development rather than a single microRNA [22]. Another promising approach to analysis and diagnosis of high-grade astrocytoma may be the investigation of ribosome synthesis machinery and parts of the nuclear genome involved in it, especially in ribosomal DNA. The instability of rDNA repeats and alterations in rRNA transcription and maturation implicates the development of many human diseases connected with poor ribosome synthesis and, thus, reduced protein production capacity or, conversely, with overexpression of proteins due to increased ribosome biogenesis in different cancers [23]. Sequence alterations in rDNA repeats and their copy number variations have been shown to correlate with high proliferation rates in tumors of different origins as well as high rRNA production and ribosome biosynthesis in cancer cells [24,25]. These observations can also be explained by the increased activity of RNA polymerase I [26], and the fact that rRNA processing and splicing pathways are altered in malignant cells [27]. In addition, human rDNA is known to be regulated epigenetically, and alterations in CpG methylation and histone modification have been shown to upregulate rRNA synthesis in glioblastoma [28,29].

Based on the fact that high proliferation rates of cancers are associated with augmentation in protein biosynthesis, recent data suggest that the nucleolus and nucleoli-associated molecules, including regulatory ones, ribosomal proteins, rDNA and ribosomal RNAs, are possible markers of cancer and promising targets of anticancer therapy [30].

## 2. Results

### 2.1. An Alu-Repeat Neighboring Region May Act as a Promoter

**Figure 1:**
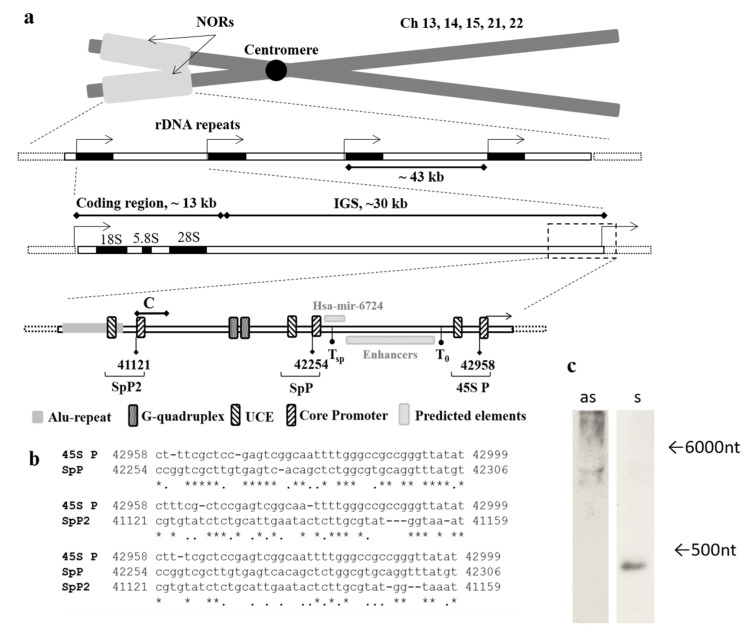
Mapping of the region of interest and its characteristics. (**a**) The schematic representation of rDNA repeats located in the p-arms of human acrocentric chromosomes within nucleolar organizer regions (NORs). Each rDNA repeat consists of coding region for 18S, 5.8S and 28S rRNAs, and ribosomal intergenic spacer (IGS). The region of interest lies ~2 kb upstream of the promoter of ribosomal DNA. C corresponds to the amplicon of C primer pair, T0—transcription termination factor I (TTFI) binding site, Tsp—predicted TTFI binding site for spacer promoter [11], SpP and SpP2—predicted spacer promoters, 45S P—rRNA gene promoter, UCE—upstream control element (UBF binding site), Core Promoter—RNA pol I binding site, Predicted elements (Hsa-mir-6724 and Enhancers) are marked according to published data [8,11]. (**b**) Alignment of core parts of 45S rRNA promoter and predicted spacer promoters SpP and SpP2, * indicates the matching nucleotides in sequences, and indicates purine or pyrimidine nucleotides. (**c**) Northern blot of total RNA from HEK293 cell line hybridized with the ABC probe for sense transcript (as) and for antisense transcript (s), the arrows indicate the length of transcripts.

Attempting to find the possible promoter upstream of the transcription start site of ribosomal genes we performed the alignment of the 2 kb region in the 40 to 42 kb region of rDNA (U13369.1) to the known 45S promoter and the predicted spacer promoter [11]. This bioinformatic analysis revealed sequence homology between the predicted spacer promoter, the core promoter of 45S rRNA gene, which lies ~2 kb upstream of the latter one, and the region 41121–41159 of rDNA (U13369.1, Figure 1a,b). It is flanked by the Alu-repeat and multiple microsatellites on the 5′-end and contains tri- and tetranucleotide repeats combined in clusters.

**Figure A1:**
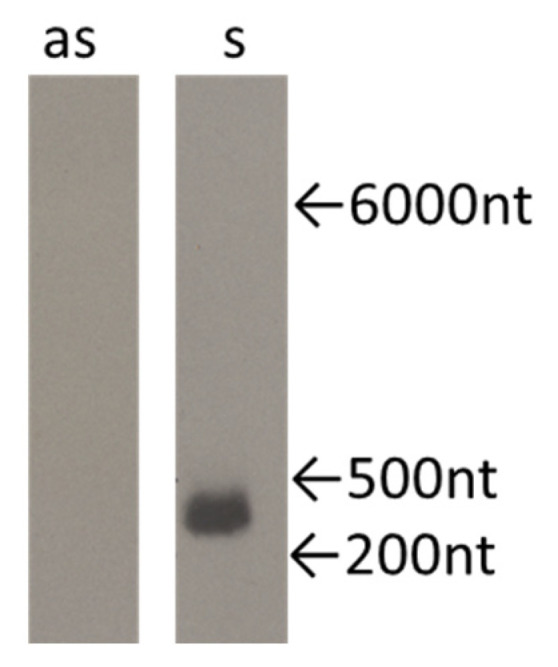
Northern blot of total RNA from HEK293 cell line hybridized with the BCD probe for sense transcript (as) and for antisense transcript (s), the arrows indicate the length of transcripts.

Using the primers designated Af and Cr (Table A1) we designed ~600 bp probes for sense and antisense transcripts covering the region corresponding to the core part of the predicted additional spacer promoter (SpP2) and the Alu-repeat. Northern blot analysis of the total RNA from HEK293 showed the hybridization of the probe with the antisense transcript 300–400 nucleotides in length, and no sense transcripts were found (Figure 1c, Figure S1, Figure A1, Figure S4).

### 2.2. The Region Upstream of the rDNA Promoter is Differentially Expressed in Cells

**Figure 2:**
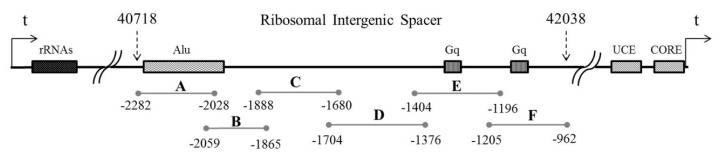
The scheme of the region under investigation with the amplicons of primer pairs designated A through F, all coordinates are given in base pairs relative to the transcription start site of 45S rRNA. rRNAs is the region of rDNA coding for 18S, 28S and 5.8S rRNAs, Alu is the Alu repeat proximal to the promoter of the downstream cluster, Gq is G-quadruplex-like structures, UCE is Upstream control element, CORE is rDNA Core promoter, transcription start site is indicated with an arrow and “t”.

**Figure 3:**
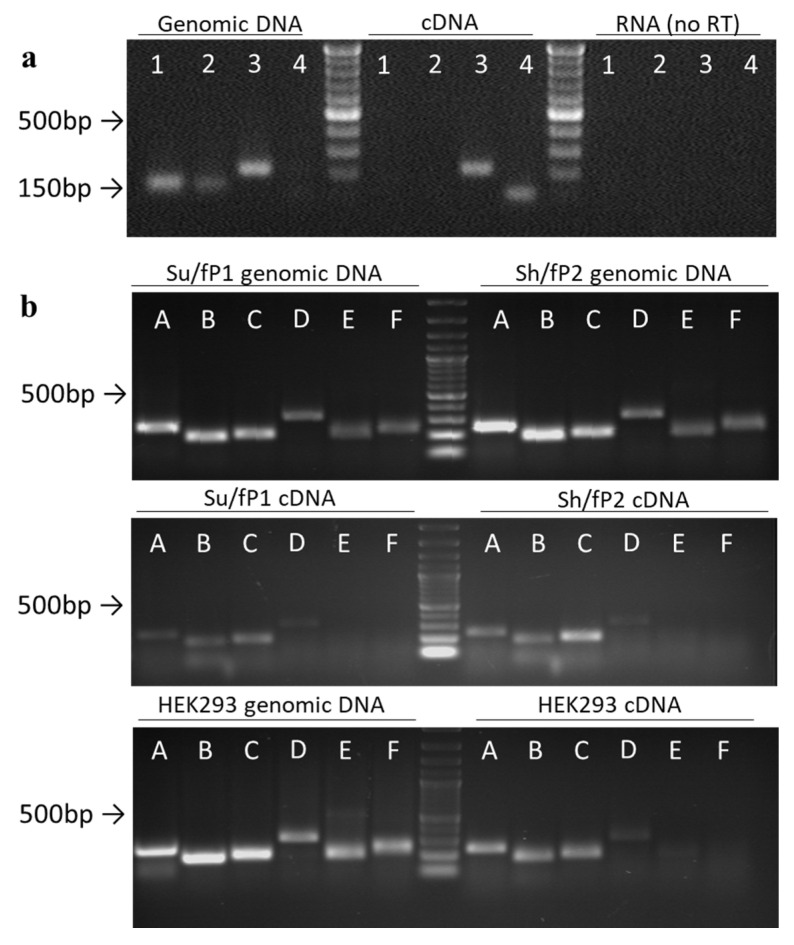
Semi-quantitative analysis of the expression of the region of interest. (**a**) Primer test on genomic DNA and cDNA from HEK293 cell line: 1–IGS36, 2–IGS42; 3–C; 4–GAPDH2 (designed to amplify cDNA only). (**b**) PCR test of primer pairs designated A through F. The sizes of bands can be compared to 1 kb DNA ladder.

**Figure A2:**
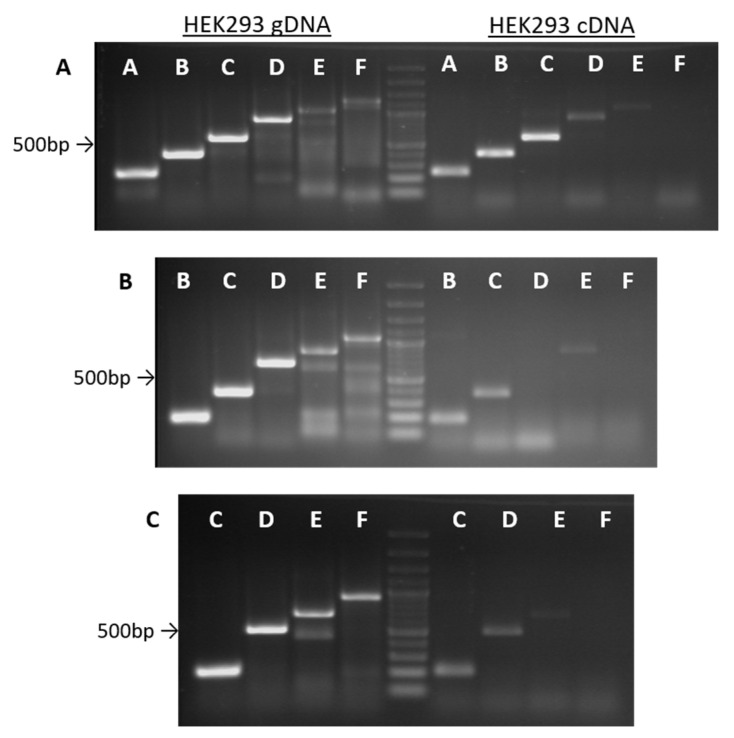
Semiquantitative primer test on HEK293 genomic DNA (gDNA) and complementary DNA (cDNA). (**A**) Amplicons obtained with A forward primer and corresponding reverse primer indicated in each of the gel lanes; (**B**) amplicons of B forward primer and indicated reverse primers; (**C**) amplicons of C forward and corresponding reverse primer indicated in the gel lanes. Equal amounts of gDNA and cDNA were taken for each of the corresponding reactions.

To map the transcript detected in the previous experiment and to estimate its expression rate in cells, we designed primer pairs to cover the ~1300 bp region of rDNA ~2 kb upstream of the promoter of ribosomal genes (Figure 2, Table A1). We also designed primers IGS26 and IGS42 upstream and downstream of the region of interest in the 36 kb and 42 kb zones of rDNA correspondingly. According to BLAST search data, these regions are not transcribed, so during PCR with the primer pairs only genomic DNA but not cDNA was amplified (Figure 3a, Figure S2). Prior to qPCR the primer pairs A–F were tested on gDNAs and cDNAs of HEK293 cell line and Su/fP2 and Sh/fP1 cell cultures (Figure 3b, Figure S3, Figure A2, Figure S5). Interestingly, the D primer pair amplicon was less abundant in the cDNA of the samples tested than A, B, and C primer pairs amplicons. The primer pairs designated E and F showed no or very slight bands on electrophoregrams and C_T_ > 33, and were excluded from further analysis for these reasons. However, the absence of the products of expected length in cDNAs and poor products in gDNAs may be due to inappropriate conditions of the reaction including both melting temperatures and specific properties of DNA polymerase; this region of IGS also contains highly repetitive sequences, which make the primer design complicated.

The cells taken for the investigation fall into two characteristic groups: 1) healthy or relatively healthy non-cancer cells (HEK293, Human astrocytes, Neuroblasts); 2) cell cultures of high-grade gliomas: D32, N42 and S54 (WHO grade III); and S40, G01, Su/fP2 and Sh/fP1 (WHO grade IV) (Table 1).

**Figure 4:**
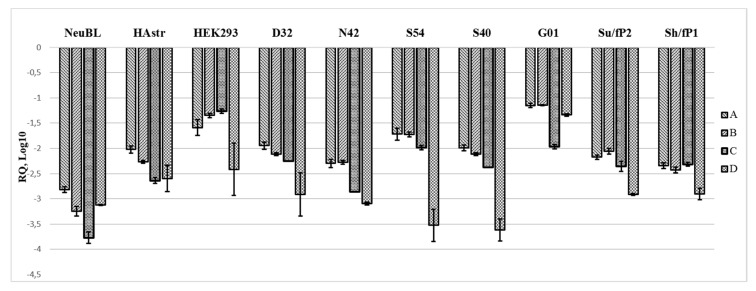
Relative quantities of the regions of interest (A–D) in different cells obtained from real-time (quantitative) PCR and normalized to the expression of GAPDH reference gene.

**Figure A3:**
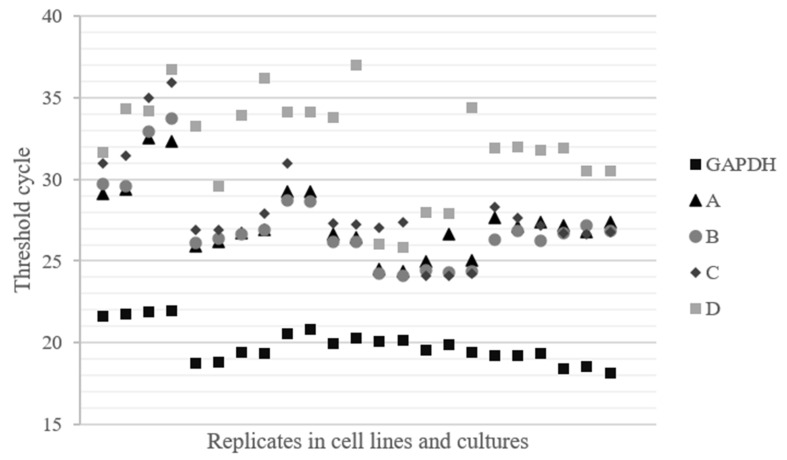
Threshold cycle (C_T_) values of GAPDH and the regions of interest in the replicates of the cell lines and cultures tested.

In all cells tested, the expression of the four regions (A, B, C, D) in the pre-promoter region normalized to glyceraldehyde 3-phosphate dehydrogenase (GAPDH) is far below what could be expected from highly repetitive sequences of rDNA, and, at first glance, demonstrates a differential expression profile (Figure 4, Figure A3). The D region seems to have the lowest expression in cells (except for G01 cell culture), which is consistent with the previous results obtained by semi-quantitative PCR. The lowest level of A, B, and C regions was detected in human neuroblasts, while in HEK293 and G01 it is much higher. In the grade IV glioma cell culture designated G01, the regions A to D have no more than a 10-fold difference between each other, but are 80 to 200-fold more expressed in this cell culture compared to the others. The expression profile of the A–D regions is similar across the cell cultures with less than a 10-fold difference in the expression of the A–C regions within the sample. The expression of region A differs 50-fold between its maximum level in G01 and minimum level in neuroblasts, while region D is expressed 200-fold higher in G01 than in S40. Neuroblasts, human astrocytes and grade IV glioma cell cultures G01, Su/fP2 and Sh/fP1 show small fold difference in the expression of the A, B, C, and D regions. The expression of the latter is no more than 10-fold lower than other regions in these cell cultures. Among them one can also note the relatively low expression of C regions compared to A, B, and D regions in neuroblasts, astrocytes and G01. In other cells (S40, S54, and HEK293) the fold difference between the expression of the D region and other regions of interest differs from 15 to 70 times, while region C is on the same level as regions A and B.

**Figure 5:**
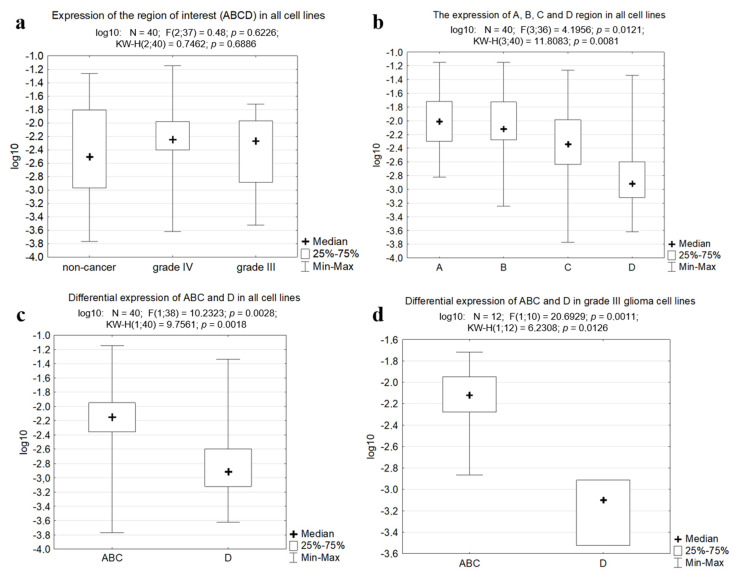
The boxplots obtained with the Kruskal–Wallis test and Statistica software. (**a**) Expression of the fused region of interest (ABCD) in all cell lines tested. (**b**) Expression of A, B, C and D regions taken separately in all cell lines tested. (**c**) Differential expression of the fused ABC region and the region D in all cell lines. (**d**) Differential expression of the fused ABC region and the region D in grade III glioma cells.

We proposed two questions which may help to estimate the results and give a possible explanation for the phenomena observed: 1) whether the expression of the region of interest (ABCD) is higher in cancer cells than in non-cancer cells; 2) whether the ABC and D regions are differentially expressed in cancer and non-cancer cells. To answer these questions, we applied statistical analysis using the Kruskal–Wallis test for multiple independent samples. The analysis of the expression of ABCD region as a whole revealed that there is no significant difference between cancer and non-cancer cells (Figure 5a), and the expression of regions A, B, C and D tested separately did not differ significantly between the groups of non-cancer and cancer cells. On the contrary, there was a significant difference (*p* = 0.0081) between the expression of each of the regions in all cells tested (Figure 5b). When we compared the expression of fused ABC regions to that of the D region, it was found to be significantly different (*p* = 0.0018, Figure 5c). Further analysis by groups revealed that regions ABC and D differed significantly only in grade III glioma cell cultures (*p* = 0.0126, Figure 5d) but not in grade IV gliomas and non-cancer cells (*p* > 0.05).

Based on these results, we suggest that the pre-promoter zone containing the regions of interest may be transcribed as a long precursor molecule which processes giving rise to at least two transcripts, one consisting of A to C regions, and the other containing the additional D region. On the other hand, the D region as a part of the transcript may form a secondary structure preventing reverse transcription and hence detection by PCR.

### 2.3. Both Sense and Antisense Transcripts are Synthesized

**Figure 6:**
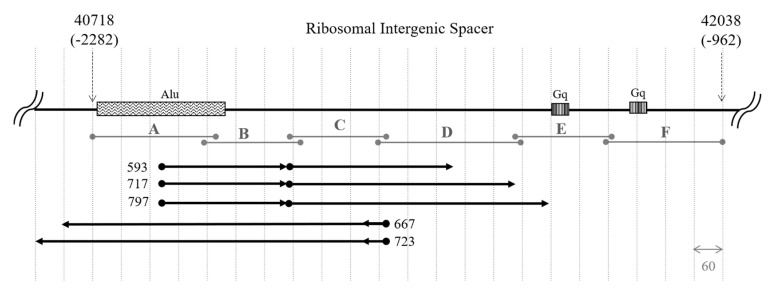
Scheme of the region under investigation from 40,718 to 42,038 of human rDNA repeating unit from transcription start site (TSS), numbers in brackets indicate the location of the region upstream of the TSS. Alu—Alu-repeat, Gq—G-quadruplex like sequences; A–F designate amplicons of corresponding primer pairs. Black arrows depict sense and antisense transcripts assembled from the results of 5′ and 3′ Rapid amplification of cDNA ends (RACEs).

To test the hypotheses mentioned above, we performed step-out PCR (3′ and 5′ Rapid amplification of cDNA ends (RACEs)) and used probes covering ABC region (A600) and CDEF region (F900), so that the transcripts synthesized from forward as well as those transcribed from the reverse strands could be detected. At first glance, the results obtained are inconsistent with the data from previous experiments: both 3′ and 5′ RACEs revealed the presence of sense and antisense transcripts in the HEK293 cell line (Figure 6). Three transcripts of different length were demonstrated to be synthesized from the bottom strand and there were two transcripts originating from the top strand. During 5′ RACE we managed to find single variants of 5′ ends in sense and antisense transcripts, while 3′ RACE gave more variety in length in both cases. The fact that 3′ ends showed more heterogeneity in length than the 5′ ends, in both sense and antisense transcripts, gives us the opportunity to predict that the sense and antisense RNAs are processed from the same corresponding precursor molecules. It may also explain the differential expression of the ABC and D regions obtained in the qPCR analysis. Additionally, the overlapping of sense and antisense transcripts in the B and C regions may contribute to the higher levels of their expression in cells. On the other hand, the phenomena whereby the transcripts’ lengths range from 667 to 723 nt for antisense transcripts, which differs from the antisense transcript length of 300–400 nt predicted during northern blot analysis, as well as the presence of three 593 to 797 nt sense transcripts absent in the northern blot, cannot be explained so far.

### 2.4. Database Analysis

**Figure 7:**
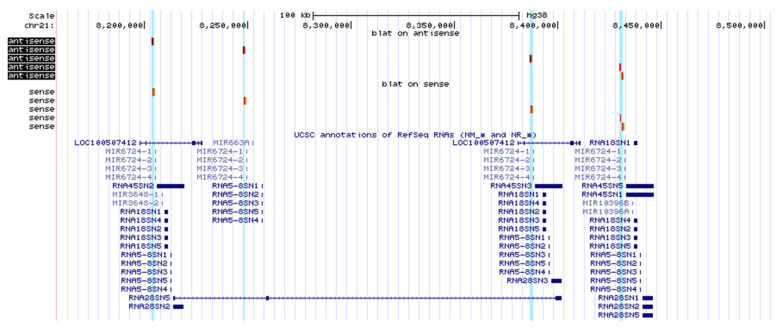
Schematic representation of human chromosome 21 region 8,158,013–8,504,512 in UCSC Genome browser. Antisense and sense transcripts sequences obtained from RACEs and aligned to the genome are shown in red and lightened in blue.

Attempting to explain the results of the experiments, we performed a BLAT-search of the sequences of sense and antisense transcripts obtained from RACEs using the UCSC (University of California, Santa Cruz) Genome Browser on Human (hg38), and they were shown to align with four zones on human chromosome 21 corresponding to 45S rDNA clusters located on its p-arm (Figure 7). Two of the zones overlap with the gene LOC100507412 (NT_167214.1, 29,568 bp), whose exons, located mostly in the 5′ and 3′ ends, code for a long non-coding RNA (NR_038958, 2945 nt). The gene transcript also includes 45S rRNA (NR_046235.3, 13,357 nt) located in the intron, which gives rise to 18S, 5.8S and 28S rRNAs. Additionally, two microRNAs (MIR6724 and MIR3648) originate from the same intron. All transcripts produced are in the sense orientation. The first 3000 bp on the 5′-end of the gene correspond to nucleolar organizing regions (NORs, MG910337), and the following 300 bp are homologous to the A region under investigation (namely, the Alu-repeat and the predicted pRNA). The further 3000 bp are aligned with NOR distal junction (MG910341) and with the annotated origin of replication in the human ribosomal RNA genes (LOC107403080, NG_046806). The remaining 22 kb of the gene coincide with the region of interest starting from the BCDEF regions and include the whole 2 kb pre-promoter zone, external and internal transcribed spacers and rRNA-coding sequences (–2 kb to 20 kb, according to U13369). Taking into account these data and the results of our experiments, we predict that the ~30 kb non-coding RNA originating from LOC100507412 may give rise not only to rRNAs, microRNAs and validated lncRNA, but also to the sense transcripts whose expression we demonstrated in the cell lines and cultures under investigation.

It is important to note, that according to the UCSC Genome Browser analysis of gene expression in 53 tissues from GTEx (The Genotype-Tissue Expression) RNA-seq of 8555 samples/570 donors and in 33 TCGA (The Cancer Genome Atlas) Cancer tissues (GENCODE v23), the gene products mentioned above are differentially expressed in normal and cancer cells. For example, long ncRNA (NR_038958) demonstrates low expression in healthy brain tissues (below 0.9 transcripts per million (TPM), while in brain lower grade glioma and glioblastoma multiforme its expression level is higher (3.2 and 4.4 TPM correspondingly).

## 3. Discussion

The high level of transcriptional activity in stress conditions of the rDNA intergenic spacer was demonstrated earlier in different human cell lines [7,8], and the promoter region of murine rRNA coding genes was shown to be transcribed, resulting in the regulatory RNA [9]. Though part of the human 45S pre-promoter region was shown to be expressed in lung cancer cell lines [13], little is known about the expression profile, role, and functions of the zones laying 2 kb upstream of the promoter sequence. Computational analysis of this region shows multiple annotated long and short, mostly non-coding RNAs. Transcription factors binding sites and promoter-like structures can also be found.

In this study, we predicted that the 2 kb upstream promoter region of human rDNA harbors the additional spacer promoter, and this zone was shown to be transcriptionally active in non-cancer cells and high-grade brain tumor cell cultures. The results obtained with quantitative PCR and subsequent statistical analysis of the data demonstrate that the expression of the parts of the transcript designated in our study as A, B, C, and D taken separately or fused together do not differ significantly in all cells tested. ABC and D regions were shown to be differentially expressed in grade III glioma cells, but not in grade IV glioblastoma cell cultures or healthy cells. One of the possible explanations of the phenomenon observed consists in the fact that all glioblastoma cell cultures had wild-type isocitrate dehydrogenase 1 (IDH1) gene, while glioma cell cultures taken for the investigation occurred to be IDH1-mutant. The mutation in this gene is characteristic to a wide range of diffuse gliomas and secondary glioblastomas [31], being a biomarker of positive prognosis and an increase of the survival [32,33]. On the one hand, mutant IDH1 ceases genetic instability of a tumor, on the other hand, some mutations in the IDH1 gene are known to upregulate DNA repair through epigenetic mechanisms and thus increase cells’ resistance therapy [34]. We are now inspired to investigate, whether the differential expression of the pre-promoter region of the IGS correlates with the mutation of IDH1 gene, or whether it can serve as additional biomarker of grade III glioma.

Another explanation that we propose is that cells produce a long transcript consisting of four regions (ABCD) that may undergo processing to form more transcripts containing the ABC regions and variable-in-length D regions in cells under some special conditions or for endogenous reasons. In this case, the following degradation of the D region decreases its relative quantity in a sample, while the ABC transcript is more stable and detected via RT-qPCR. This is supported by the fact that the transcripts originating from IGS indeed possess different lifetimes [35]. It is also possible that the D region is not degraded but forms a secondary structure preventing reverse transcription, which could explain its expression pattern in glioma grade III cell cultures. In any case, the fact that the ABC and D regions are differentially expressed in glioma grade III, but not in glioblastoma grade IV, and non-cancer cells also hints at the observation that highly malignant tumor cells may mimic normal ones to avoid detection or disruption by heathy cells of the body.

During the analysis of total RNA isolated from the HEK293 cell line taken as a model, antisense transcripts were demonstrated using northern-blot analysis, and step-out PCR (RACE) methods revealed the presence of both sense and antisense transcripts of different lengths that overlap. These controversial results may be explained by the fact that the number of antisense transcripts is relatively high compared to sense transcripts. Nevertheless, it is important to note that, among the annotated RNA molecules homologous to the sequences obtained in our investigation, there were only sense transcripts. On the one hand, the overlapping of the sense and antisense transcripts in the ABC regions may contribute to the higher expression observed during qPCR analysis. On the other hand, it may result in an unusual intermolecular RNA secondary structure, which leads to the silencing of each of the molecules, or, conversely, gives rise to some new regulatory RNAs. In many cases, it is the secondary structure, not the sequence of RNA, which determines its effects [32]. For example, the pRNA originating from the pre-promoter region of murine rDNA was shown to be processed from a ~2 kb precursor and to regulate not only the expression of ribosomal RNAs but also the formation of heterochromatin [9]. Cell differentiation based on this mechanism involves both the hairpin formed by the pRNA and the precursor IGS-rRNA, which impairs the recruitment of the silencing complex guided to the promoter by the former molecule, while the mature pRNA stimulates cell differentiation, suggesting that its processing is the key process in heterochromatin formation [36].

In the Mus musculus, IGS-rRNA is transcribed from the spacer promoter, which was mapped using deconvolution ChIP-Seq. Although human and murine rDNA sequences differ significantly in the IGS region, the organization of the promoter sequences seems to be very alike. The human spacer promoter located between −850 and −700 bp relative to the transcription start site has homologous sequences with the rDNA promoter. Moreover, it can be regulated in a similar manner by binding of Transcription Termination Factor I [11].

Taken together, the data obtained suggest that the transcripts revealed in this study may possibly have a secondary structure which determines their functions like other non-coding RNAs transcribed from the IGS [7,9]. For example, they may participate in the regulation of the spacer promoter by guiding silencing complexes to it when regulatory RNA is processed or impairing their formation in the opposite case. Interestingly, the sequence downstream of the potential spacer promoter codes for the microRNA hsa-mir-6724 predicted earlier, which can have targets among mRNAs coding for proteins associated with cancer development pathways [37].

Though the current work did not suggest any revealing of the functions of the transcripts detected, having the particular sequences we are now able to determine their protein counterparts, which may help to expand our knowledge and understanding of the molecular mechanisms by which rDNA and its non-coding regions contribute to the proper regulation of the whole genome functioning described below.

Ribosomal DNA is differentially expressed during cell growth and maturation, and the silent copies were shown to influence the heterochromatin formation and induce cell differentiation [38]. The actively transcribed pre-promoter region of the IGS may possess a key role in this process by producing short regulatory non-coding RNAs which are capable of attracting chromatin-remodeling complexes and enzymes both to rDNA and topologically close chromosomal domains [39]. If the process of heterochromatin formation in the rDNA genes is disrupted, a large number of double strand breaks (DSBs) appear in this region, leading to genomic instability [40]. Damage such as DSBs within rDNA induce a large-scale nucleolar reorganization and recruitment of the homologous repair machinery independently of the stage of cell cycle [41]. While silent rDNA protects the genome from damage and excessive recombinational events, actively transcribed rRNA genes contribute to the regulation of replication [42]. Blocking of replication forks in the region of ribosomal RNAs genes leads to the activation of repair processes in which rDNA has a major initiator role, and the disruption of the mechanism of rDNA-dependent conducting of DNA repair leads to the inducement of repeat instability [43]. Altogether, ribosomal RNA coding regions in the human genome tend to be extremely unstable: the copy number of 45S rRNA genes may change with aging [44] and under different conditions [45], it differs in healthy individuals and patients with a disease [46], and can be both increased and decreased in cancers [25]. Induced by improper DNA repair, rDNA copy number variation is associated with global reorganization of gene expression and may contribute to cell’s adaptation abilities [47].

The results of our study also support the recently obtained data indicating that rRNA gene clusters differ not only in the number of genes within the genome and between the individuals but also in their molecular content, i.e., sequence, regulatory elements, protein binding sites, etc [48]. We are unaware of the particular functions of the non-coding RNAs mapped in the pre-promoter region of rDNA, yet taking into account their molecular context and its variability, the possible roles in the key cellular processes such as gene expression regulation, heterochromatin formation, genomic instability induction and DNA repair initiation cannot be underestimated [49].

Another fundamental question raised by this study’s data applies to the fact that little is known about the regulation of transcription of multiple non-coding RNAs originating from the IGS, and the results to date seem to be controversial. Despite the fact that RNA pol I was shown to be associated with the rRNA coding regions and their promoter but not with the intergenic non-coding sequences [50], it was demonstrated to transcribe 10 kb long non-coding RNA from the IGS [51]. In yeast RNA pol II transcriptional activity was demonstrated in the rDNA non-coding regions [52], and the pol II derived transcripts were shown to regulate nucleolar dynamics in higher eukaryotes [53]. In addition, the ribosomal intergenic spacer contains multiple Alu-repeats, evolutionarily originated from 7SL RNA genes [54], and may be potentially transcribed by RNA pol III [55]. We further plan to determine both the enzyme involved in the transcription of the non-coding RNAs detected in the current study and regulation of their expression.

To sum up, despite the fact that rDNA is a rather conserved genome region, its non-coding parts, namely intergenic spacers, differ between species, individuals, cells, and even within the same cell. Regulatory sequences within the IGS participate in DNA replication control and recombinational repair conducting, and non-coding RNA molecules originating from the IGS orchestrate heterochromatin formation which influences the above-mentioned processes. Thus, the careful study of IGS-derived non-coding transcripts may be of great importance in understanding the mechanisms of genomic instability and DNA repair in cancer cells.

## 4. Materials and Methods

### 4.1. Cell Lines and Cultures Characteristics

The summary of cell lines and cultures taken for analysis is represented in Table 1. NeuBL are non-cultivated cells obtained from an aborted fetus. HAstr is a cell culture derived from human astrocytes obtained from an aborted fetus. HEK293 is a specific cell line originally derived from human embryonic kidney cells grown in tissue culture. Glioma (WHO grade III) cell cultures D32, N42 and S54, and glioblastoma (WHO grade IV) cell cultures S40, G01, Su/fP2 and Sh/fP1 were derived from surgical samples of patients with the diagnoses as described before [56]. All materials were provided by the laboratory of Neurogenetics and Developmental Genetics of the Institute of Gene Biology (IGB) of the Russian Academy of Sciences, Moscow, Russia. All cell cultures except NeuBL were grown in monolayer in a DMEM/F12 medium prior to total RNA and genomic DNA extraction.

### 4.2. RNA and DNA Extraction

RNA extraction was performed using RNAzol RT reagent (MRC, Cincinnati, OH, USA) according to the manufacturer’s instructions for isolating total RNA. Additionally, RNA samples (~15 µg) were treated with 3 units of DNase I (Thermo Fisher Scientific, Vilnius, Lithuania) with the addition of 40 units of RiboLock RNase Inhibitor (ThermoFisher Scientific, Lithuania) for 40 min at 37 °C in an appropriate buffer solution and then precipitated in isopropanol after phenol-chloroform extraction. Precipitants were dissolved in nuclease-free water and used in downstream analysis or stored at −70 °C. The purity of isolated RNA samples was tested using PCR analysis with 18S primers (Table A1).

Genomic DNA extraction from cultured cells was performed using phenol-chloroform extraction prior to which cells harvested using Trypsin-EDTA were lysed in a lysis buffer (1% sodium dodecyl sulfate (SDS), 100 mM Tris-HCl pH 8.0 and 50mM EDTA pH 8.0) and incubated at 37 °C at least for 2 h with Proteinase K (Promega, Madison, WI, USA).

### 4.3. Northern Blot Analysis

An aliquot of total RNA dissolved in 50% formamide with the addition of x2 loading dye (Ambion, Carlsbad, CA, USA) was applied to denaturing electrophoresis in a formaldehyde running buffer (1 M MOPS, 0.5 M sodium acetate, 50 mM EDTA, 2.2 M formaldehyde). Capillary transfer to a positively charged nylon membrane (Roche, Mannheim, Germany) was performed in a transfer buffer (3 M NaCl, 10 mM NaOH) for 2 h with the following covalent linkage to the membrane by 254 nm UV light for 50 sec. Biotinylated RNA probe was synthesized from A600 and F900 plasmids (described in Southern-blot analysis) using T7 RNA Polymerase-Plus™ Enzyme Mix (Ambion, USA) and Bio-16-UTP (Ambion, USA) according to the manufacturer’s instructions. Hybridization was performed in an UltraHyb Ultrasensitive Hybridization Buffer (Ambion, USA) at 68 °C overnight. Then the membrane was washed in the washing buffer (0.1% SDS and 0.1× SSC (Saline Sodium Citrate buffer), a TBS-T buffer (0.2 M Tris, 1.5 M NaCl, 0.05% Tween-20) and incubated in a maleic buffer (100 mM Maleic acid, 250 mM NaCl, pH 7.5) containing 10% Blocking reagent (Roche, Germany) at room temperature for an hour, then 1.5 μL of Pierce Streptavidin Poly-HRP (ThermoFisher Scientific, Lithuania) per 20 mL blocking solution was added and the membrane was incubated at room temperature for an hour. After subsequent washing in TBS-T, detection was performed using the Amersham ECL Prime Western Blotting Detection Reagent (Merck KGaA, Darmstadt, Germany) and developing proceeded with Amersham Hyperfilm ECL (Merck KGaA, Germany).

### 4.4. Primer Design

Six primer pairs covering the region of interest in the pre-promoter zone of rDNA were designed with the help of Primer-BLAST [57] to anneal at ~60 °C and elongate, resulting in ~200 bp products (Table A1, Figure 2) in the pre-promoter region of rDNA (according to the GenBank sequence U13369.1, https://www.ncbi.nlm.nih.gov/nuccore/555853). Before applying primer pairs to cDNAs and qPCR, they were tested using PCR on gDNA of several cell cultures as a template. Polymerase chain reaction was performed with 2 µL of 10X Buffer and 2 µL of MgCl2 (Thermo Fisher Scientific, Lithuania), 100 ng of template, 2 µL of 2 mM primers, an aliquot of Taq Polymerase (Thermo Fisher Scientific, Lithuania) and water to the volume of 20 µL. The run protocol consisted of 30 cycles of 95 °C for 15 s, 60 °C for 15 s, and 72 °C for 30 s. Amplicons were also confirmed by sequencing. Additional primer pairs were used in the experiment for positive and negative controls.

### 4.5. Primer Efficiency Determination

Reaction products after gDNA amplification with all primer pairs used in the subsequent qPCR analysis of gene expression were cloned into a pGEM-T Easy vector (Promega, USA) for determination of primer efficiencies with the help of a standard curve method performed on an AB StepOnePlus Real-Time PCR system (Thermo Fisher Scientific, Carlsbad, CA, USA) using SYBR Green reagent qPCRmix-HS SYBR + HighROX (Evrogen, Moscow, Russia) and analyzed by means of StepOne Software v.2.3 (R2 > 0.991 for all primer pairs and efficiencies 70–90%). Primer efficiencies used in the further estimation of fold difference of target sequences expression with delta CT method were calculated as follows (1):

(1) $$E=10^{\left(-\frac{1}{a}\right)},$$

where E is a primer pair efficiency and a is the slope obtained from the standard curve.

### 4.6. Reverse Transcription and qPCR

Reverse transcription was performed using an MMLV RT kit (Evrogen, Russia). Moreover, 2 µg of total RNA, 1 µL of random hexamer primer, 1 µL of DTT, 1 µL of dNTPs, 1 µL of MMLV RT and nuclease-free water to the volume of 10 µL were mixed and incubated at 42 °C for 60 min.

The comparative CT experiment was performed on an AB StepOnePlus Real-Time PCR system (Thermo Fisher Scientific, USA) using SYBR Green reagent qPCRmix-HS SYBR + HighROX (Evrogen, Russia) with the reaction set up according to the manufacturer’s instructions. All reactions were performed in duplicates or triplicates including negative controls in which RNA was added instead of the template. The run protocol consisted of 40 cycles of 95 °C for 15 s, 60 °C for 15 s, and 72 °C for 30 s. Fluorescence intensity was measured after the elongation stage. A melt curve analysis was conducted to confirm the specificity of PCR products. The expression of target sequences was normalized to that of the glyceraldehyde-3-phosphate dehydrogenase (GAPDH) reference gene, and the fold difference of expression (RQ) was calculated using the following Equation (2):

RQ = E^−ΔC^_T_, (2)

where RQ is fold difference of expression, E is primer efficiency determined before, ΔCT is the difference in CT values for a target sequence and the housekeeping gene (GAPDH) for a given sample. GAPDH was chosen as a reference gene, as it showed roughly equal CT values in the cells tested (Figure 3A).

The variance of the ΔC_T_ was calculated from the standard deviations of the target and reference values using the following Equation (3):

(3) $$s=\surd \left(s_{1}^{2}+s_{2}^{2}\right),$$

where s1 is standard deviation in the expression of target sequences and s2 is that of the reference gene. The standard deviation values were <0.25 for all samples.

### 4.7. 3′ RACE

For first strand DNA synthesis ~2.5 μg of both total RNA and in vitro polyadenylated RNA were taken. For polyadenylation we used *Escherichia coli* Poly(A) Polymerase (NEB, Ipswich, MA, USA) according to manufacturer’s instructions: about 10 μg of total RNA dissolved in 15 μL H2O were mixed with 2 μL of 10× buffer, 2 μL of ATP (10 mM) and 1μL of *Escherichia coli* Poly(A) Polymerase. The mixture was incubated at 37 °C for 30 min with subsequent phenol-chloroform extraction and isopropanol precipitation.

The reverse transcription reaction for 3′ RACE was performed in the volume of 20 μL: RNA dissolved in a corresponding volume of water and mixed with 2 μL of 10× buffer, 2 μL of random primer and 1 μL of 3′ RACE adapter (Table A2) was denatured at 65 °C for 10 min and immediately placed into ice, then 2 μL of dNTPs and 2 μL of MMLV-RT (Evrogen, Russia) were added. Reaction proceeded at 42 °C for 40 min and an aliquot was then used for the first round PCR with Cr or Cf primer and 3′ RACE primer (Table A2). The reaction was performed with the use of Phusion High-Fidelity DNA Polymerase (Thermo Scientific, Lithuania) in 20 μL according to the manufacturer’s instructions for reaction set up and 3-step run protocol. Aliquots of the first round PCR were analyzed using agarose gel electrophoresis and Southern blotting. Membranes were hybridized with F900 probe for “sense” (Cf + 3′ RACE primer) and A600 probe for “antisense” (Cr + 3′ RACE primer) samples. Following hybridization, we separated the samples by gel electrophoresis and the sharpest bends showing the highest signal were extracted, A-tailed, purified and linked to the pGEM-Teasy vector. Plasmids from positive colonies were extracted and sequenced by Evrogen (Russia).

### 4.8. 5′ RACE

Total RNA was treated with rSAP (NEB, USA) with subsequent RppH (NEB, USA) treatment and ligation with a 5′ RACE adapter (Table A2) using T4 RNA ligase (Promega, USA) at 37 °C for 1 h. After each of these procedures RNA underwent phenol-chloroform extraction and isopropanol precipitation. Finally, reverse transcription was performed with M-MuLV RT (NEB, USA) according to the manufacturer’s instructions. First round PCRs were performed with Cr primer and 5′ outer primer for “sense” samples and Bf primer and 5′ outer primers for “antisense” samples (Table A2). Reactions were set as follows: 20 μL of 5× Phusion GC Buffer, 2 μL of dNTP, 2 μL of each of the primers in the primer pair, 4 μL template, 1 μL of Phusion High-Fidelity DNA Polymerase and 70 μL of nuclease-free water. The reaction mix was then separated into four tubes and each proceeded with the following run protocol with annealing temperature gradients: 98 °C for 30 s of initial denaturation and 35 cycles of 98 °C for 10 s, 62/60/58/56 °C for 10 s, 72 °C for 60 s. Second round PCRs were performed using 1 μL of previous (first round) reaction mixtures with the addition of Br primer and 5′ inner primer for “sense” samples and Cf primer and 5′ inner primer for “antisense” samples (Table A2). The reaction mixtures were prepared as before, as was the run protocol. Aliquots of second round mixtures were analyzed with gel electrophoresis, blotted and hybridized with corresponding probes (A600 for sense and F900 for antisense samples). Following the analysis, the samples underwent the same procedure as those in the 3′ RACE.

### 4.9. Southern-Blot Analysis

Probes for Southern-blot analysis used in 3′ and 5′ RACEs were obtained using PCR amplicons of Cf and Fr primer pair for the F900 probe and Af and Cr primer pair for the A600 probe. Amplicons were separated by gel electrophoresis, extracted, A-tailed, purified and linked to the pGEM-Teasy vector. After plasmids extraction and purification from positive colonies, they were confirmed by sequencing and used in probe synthesis with the help of a Biotin DecaLabel DNA Labeling Kit (ThermoSientific, Lithuania) according to the manufacturer’s instructions.

Amplicons were separated in 1% agarose gel with subsequent incubation in a denaturing buffer (0.5 M NaOH, 2.5 M NaCl) for 5 min. DNA transfer to the nylon membrane (Roche, Germany) was performed in the denaturing buffer for 2 h with the following wash in neutralizing solution (1M Tris pH 7.4, 1.5 M NaCl) and cross-link at 254 nm UV for 50 s. The membrane was incubated in a Church–Gilbert buffer (0.5 M Na_2_HPO_4_, 10 mM EDTA, 7% SDS pH 7.5) at 63 °C for 30 min, then the probe was added and the membrane was incubated with the mixture at 63 °C overnight. The subsequent steps were the same as described in northern-blot analysis.

### 4.10. Bioinformatic Analysis

The search for possible promoters and promoter-like structures as well as transcription factors binding sites in the Human ribosomal DNA complete repeating unit (GenBank: U13369.1, https://www.ncbi.nlm.nih.gov/nuccore/555853) were performed using the following online open-access internet tools: Promoter 2.0 [58], FPROM and Nsite [59], CNNPromoter [60], GPMiner [61]. The homology between the promoters found was estimated using the multiple sequences alignment tool MAFFT [62].

To search for the genome location and neighboring features of the region of interest we applied the UCSC Genome Browser [63] and BLAT [64].

Statistical analysis was performed using Statistica software (StatSoft, Moscow, Russia), according to the protocol for analysis of multiple independent groups (the Kruskal–Wallis test). This test was chosen because the three groups compared (non-cancer cells, grade III glioma, grade IV glioblastoma) are independent, and the variables compared in the groups (RQ, log10) do not demonstrate normal distribution. The difference in expression was considered significant if *p* < 0.05, otherwise the groups compared were considered to differ insignificantly.

## 5. Conclusions

Gene clusters coding for 28S, 5.8S, and 18S ribosomal RNAs draw the attention of investigators for their outstanding contribution to a cell’s protein synthesis machinery, response to stress and inducement of DNA repair, orchestration of heterochromatin formation and cell differentiation. Elucidation of the changing of rDNA structure and expression, and, in particular, the transcription and functions of its non-coding sequences—intergenic spacers—in brain tumors is of great importance both for cancer biologists and fundamental molecular biology studies.

The IGS is transcribed differentially in normal and cancer cells, and the expression is influenced by the conditions, giving rise to a variety of non-coding RNAs possessing different functions needed for the maintenance of the cell functioning. Deep investigation of the secondary structures as well as the determination of RNA and/or protein counterparts of the transcripts originating from pre-promoter regions of the IGS will contribute to our understanding of the functioning of rRNA coding genes and their role in tumor cells’ genetic instability and their development.

## Figures and Tables

**Table 1:** Cell cultures taken for analysis and their characteristics.

Healthy or Relatively Healthy Cells		Malignant Cells	
HEK293	Human embryo kidney	D32	Glioma III
Hastr	Human astrocytes	N42	Glioma III
Neubl	Neuroblasts	S54	Glioma III
–	–	S40	Glioblastoma IV
–	–	G01	Glioblastoma IV
–	–	Su/fP1	Glioblastoma IV
–	–	Sh/fP2	Glioblastoma IV

## Supplementary Materials

The following are available online at https://www.mdpi.com/2072-6694/12/8/2090/s1, Figure S1: Original blot images for Figure 1c. Areas shown in the paper in Figure 1c are highlighted here with black rectangles, Figure S2: Figure 3a original gel image, Figure S3: Figure 3b original gel images, Figure S4: Figure A1 original blot image. Areas shown in the paper in Figure A1 are highlighted here with black rectangles, Figure S5: Original gel images for Figure A2.

## Abbreviations

bp	base pairs
cDNA	complementary DNA
ChIP-Seq	chromatin immunoprecipitation and sequencing
gDNA	genomic DNA
IGS	intergenic spacer
kb	kilo base pairs
NOR	nucleolar organizer region
pRNA	promoter RNA
PCR	polymerase chain reaction
qPCR	quantitative PCR
RACE	rapid amplification of cDNA ends
sncRNA	small non-coding RNA
WHO	World Health Organization

## Appendix A

**Table A1:** Primers used in PCR and qPCR analysis. f–forward primer and r–reverse primer.

Name	Sequence (5′–3′), Orientation (f/r)	Melting Temperature, °C	Amplicon Size, bp
A	GTTTCGTCCTTTTGAGACAGAGT, f GTGGGCGCATCACAGGAGGTC, r	59 66	254
B	TCCAACTCCCGACCTCCTGT, f TGCAGAGATACACGTTGTCGTTG, r	63 62	194
C	CAACGACAACGTGTATCTCTGCA, f TACGCTCGGTTCATTTACACACA, r	62 61	208
D	ATGTGTGTAAATGAACCGAGCGTA, f TACACAAAGTAAACTTCTGAAACAC, r	61 57	328
E	GGAGTGTTTCAGAAGTTTACTTTGTG, f AGACACACGGAGAGGCAGAAT, r	60 62	208
F	CGTGTGTCTGCAGCGACC, f GAAAGCGAAACCGTGAGTCGA, r	62 62	243
18S	TTTCTCGATTCCGTGGGTGG, f CCCGGACATCTAAGGGCATC, r	60 60	211
GAPDH	CCATGGGGAAGGTGAAGGTC, f AGTGATGGCATGGACTGTGG, r	60 60	548
GAPDH2	ACCGTCAAGGCTGAGAACG, f GCATCGCCCCACTTGATTTT, r	60 59	95
IGS36	GCACCTCTCCGGAAACATTG, f CTTAACCACGCACCCACGAA, r	58 59	150
IGS42	GCGATCCTTTCTGGCGAGTC, f CGGGAGCCGGAAGCATTTTC, r	60 60	143

**Table A2:** Primers used in 3′ and 5′ RACEs (From FirstChoice RLM-RACE kit, Ambion, USA).

Name		5′–3′ Sequence
3′ RACE	Adapter	GCGAGCACAGAATTAATACGACTCACTATAGGT12VN
	Primer	GCGAGCACAGAATTAATACGACT
5′ RACE	Adapter	GCUGAUGGCGAUGAAUGAACACUGCGUUUGCUGGCUUUGAUGAAA
	Outer Primer	GCTGATGGCGATGAATGAACACTG
	Inner Primer	CGCGGATCCGAACACTGCGTTTGCTGGCTTTGATG
