# Supplementary material for: Mapping and Quantification of Non-Coding RNA Originating from the rDNA in Human Glioma Cells
Source: Cancers (Basel). 2020 Jul 28;12(8):2090. doi: 10.3390/cancers12082090 (PMC7464196; doi:10.3390/cancers12082090)
Supplement: Supplementary file 1 [file cancers-12-02090-s001.pdf]

cancers

Article

Mapping and Quantification of Non-Coding RNA Originating from the rDNA in Human Glioma Cells

Anastasia A. Sadova, Natalia S. Kupriyanova and Galina V. Pavlova

Supplementary Materials

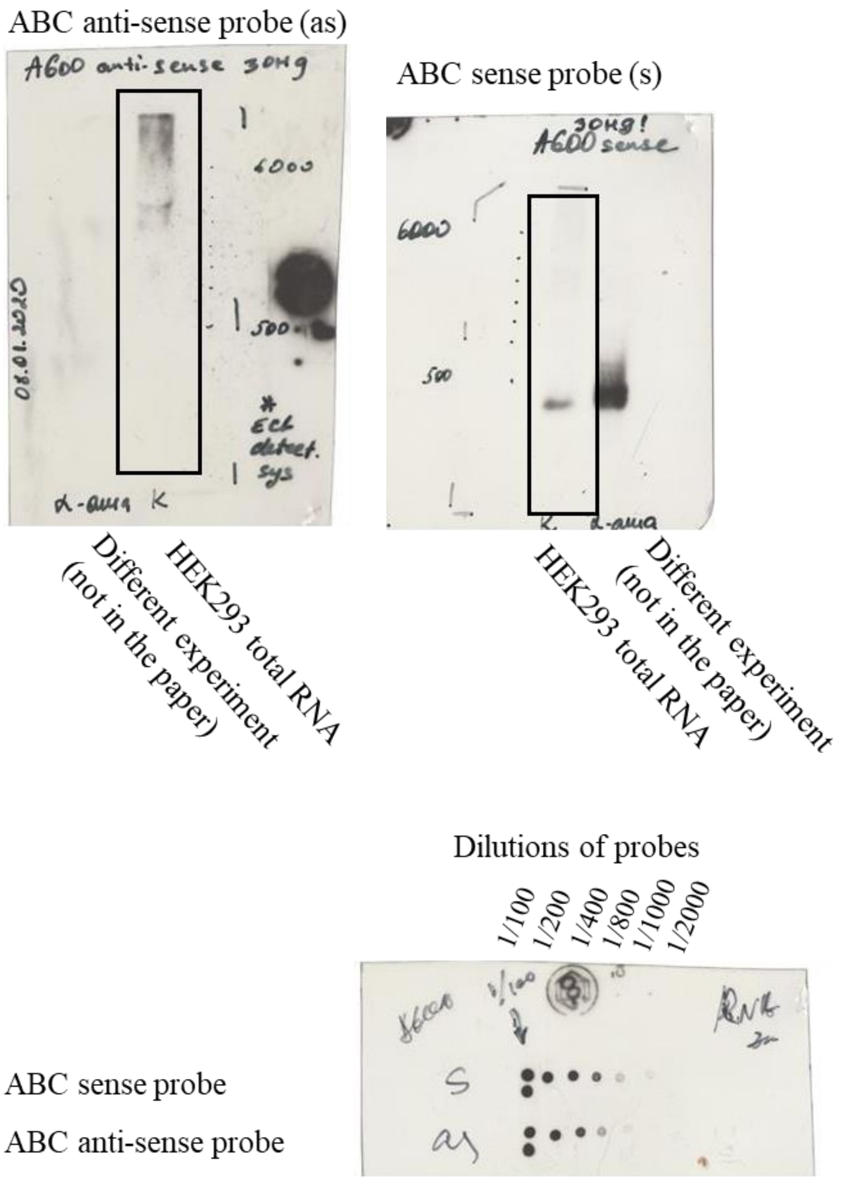

Figure S1. Original blot images for Figure 1c. Areas shown in the paper in Figure 1c are highlighted here with black rectangles.

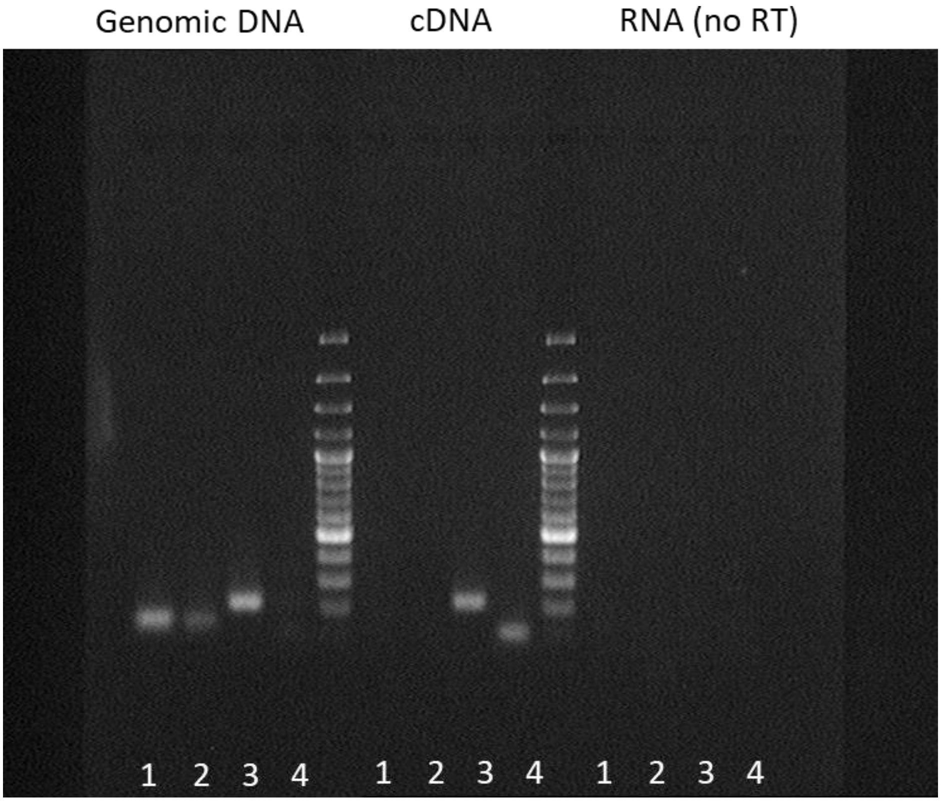

Figure S2. Figure 3a original gel image.

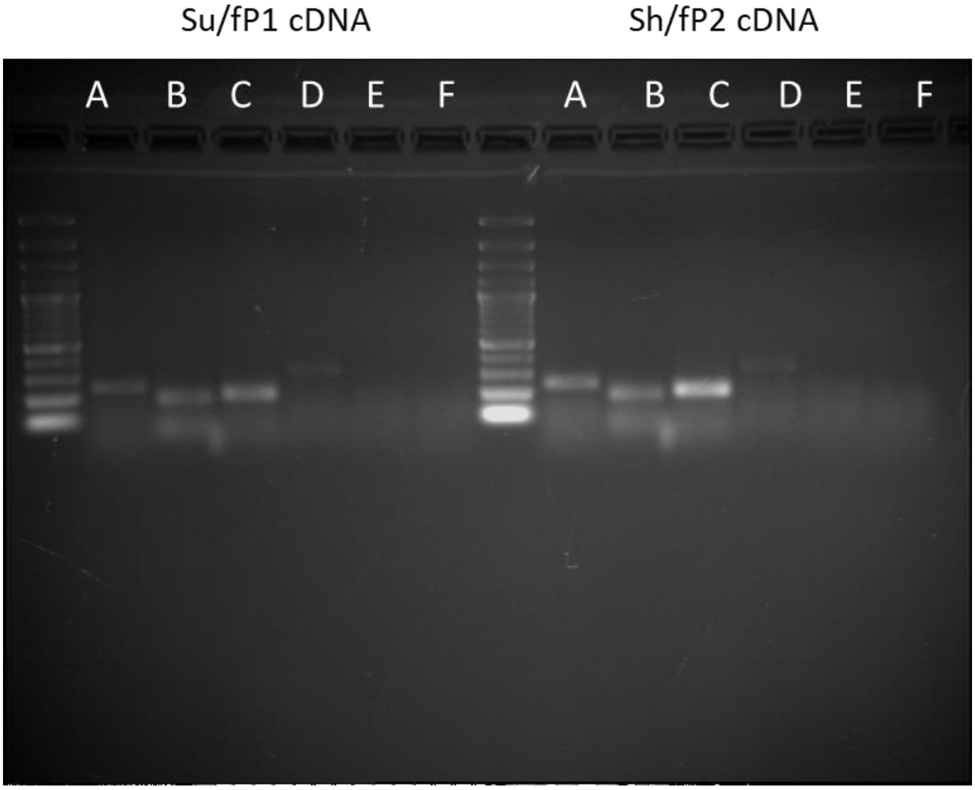

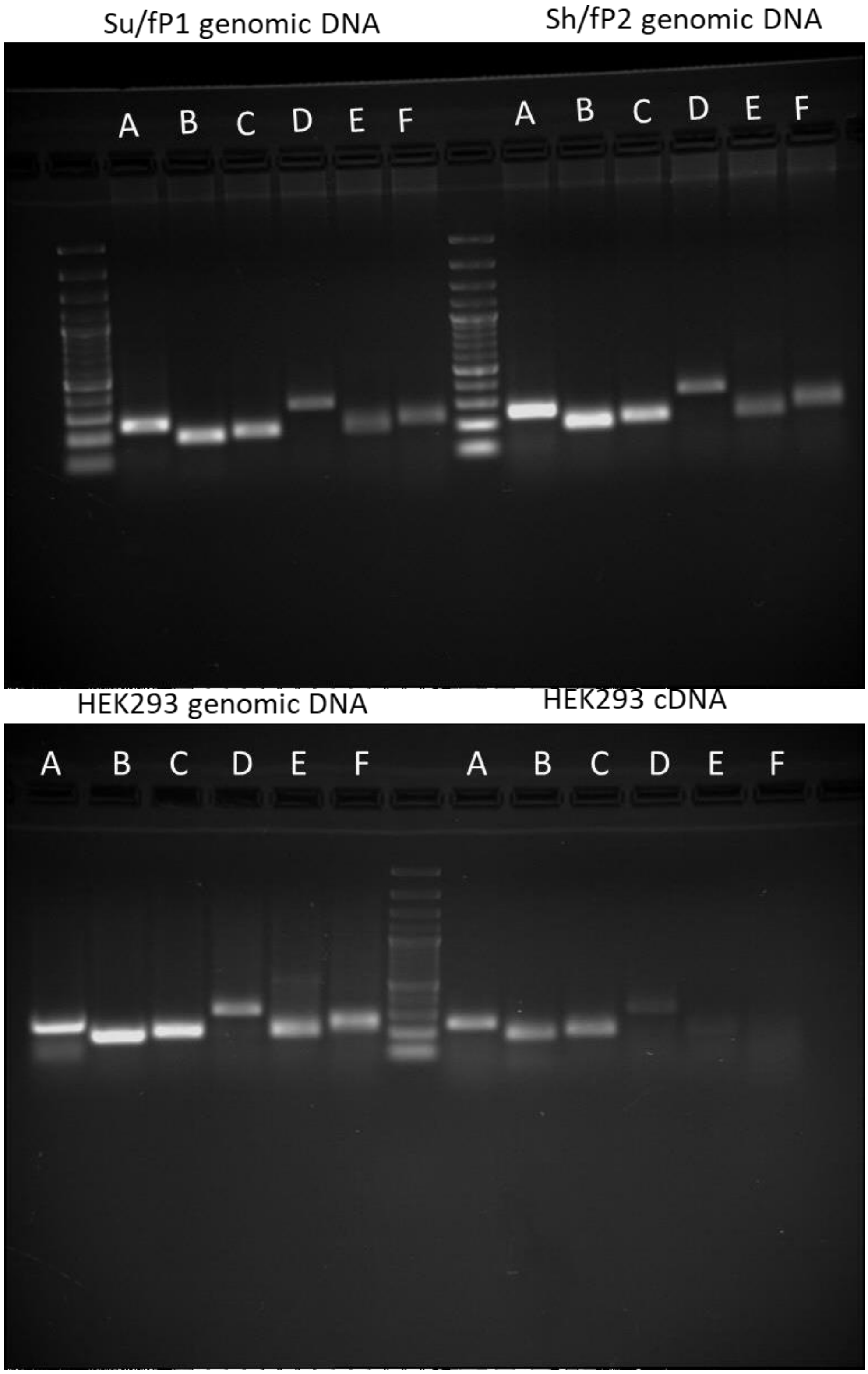

Figure S3. Figure 3b original gel images.

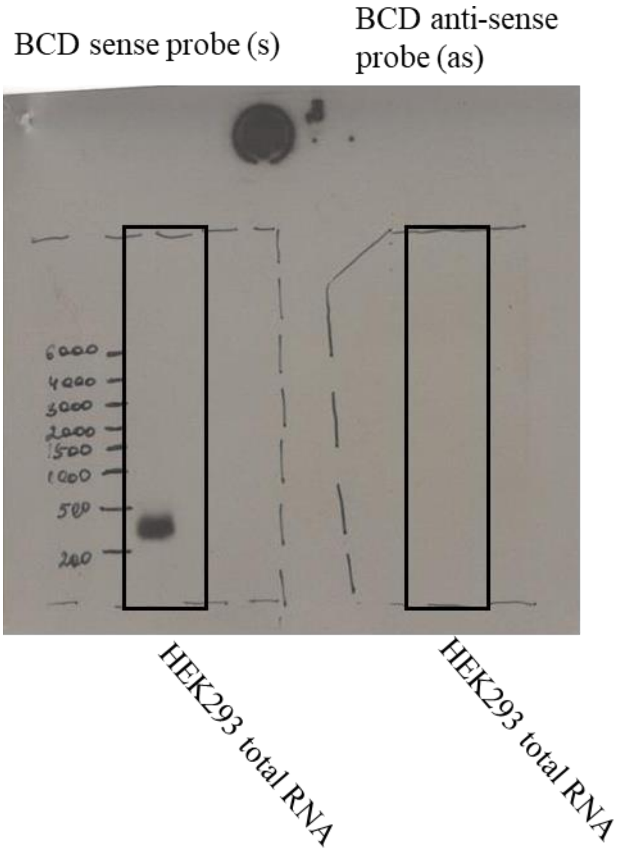

Figure S4. Figure A1 original blot image. Areas shown in the paper in Figure A1 are highlighted here with black rectangles.

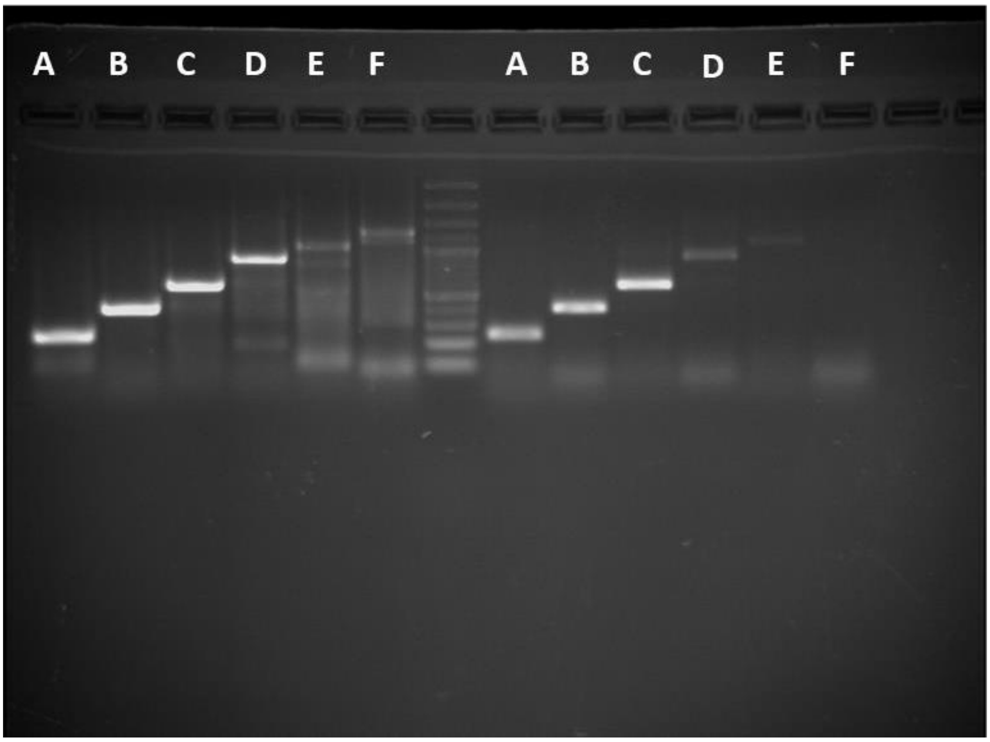

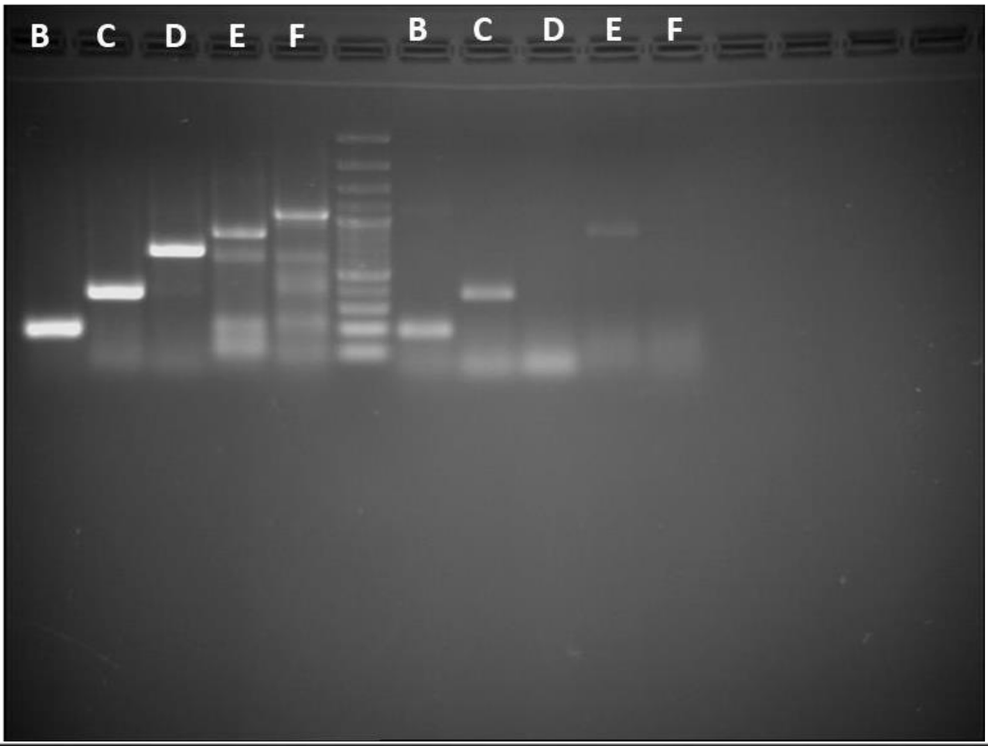

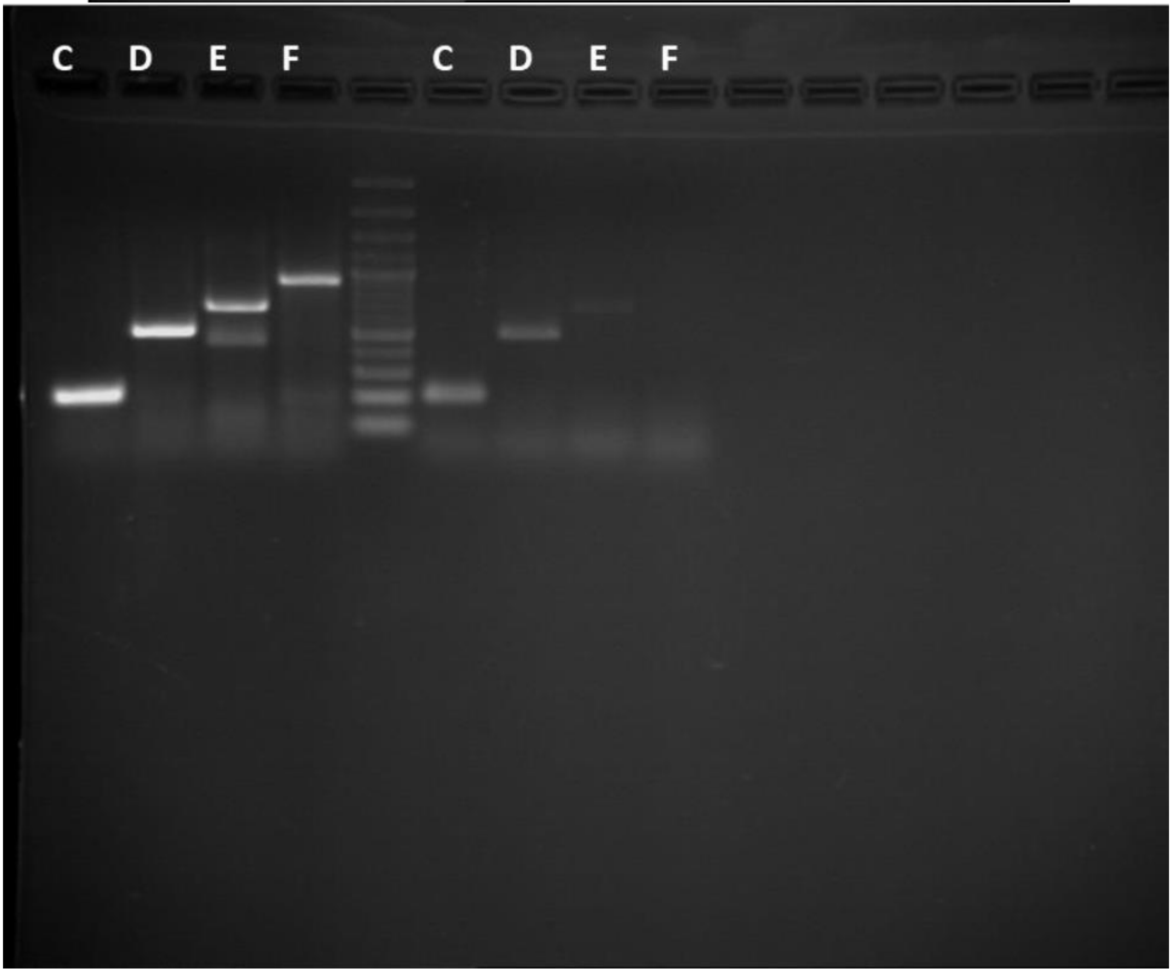

Figure S5. Original gel images for Figure A2.
